# Toxic mechanisms of amyloid oligomers and therapeutic strategies

**DOI:** 10.1002/pro.70519

**Published:** 2026-03-12

**Authors:** Magdalena I. Ivanova, Carmelo La Rosa, Ayyalusamy Ramamoorthy

**Affiliations:** ^1^ Biophysics Program University of Michigan Ann Arbor Michigan USA; ^2^ Michigan Neuroscience Institute University of Michigan Ann Arbor Michigan USA; ^3^ Dipartimento di Scienze Chimiche Università degli Studi di Catania Catania Italy; ^4^ Department of Chemical and Biomedical Engineering, FAMU‐FSU College of Engineering Florida State University Tallahassee Florida USA; ^5^ National High Magnetic Field Laboratory Tallahassee Florida USA; ^6^ Institute Molecular Biophysics Florida State University Tallahassee Florida USA; ^7^ Department of Chemistry University of Michigan Ann Arbor Michigan USA

**Keywords:** amyloid disease, neurodegeneration, oligomers, prions, protein misfolding, therapies, toxicity mechanisms

## Abstract

Amyloid oligomers are increasingly recognized as the major toxic contributors across protein‐misfolding disorders. In this review, we cover mechanistic evidence showing how these transient and structurally heterogeneous oligomers disrupt cellular homeostasis by: (i) permeabilizing lipid membranes and forming ion‐conducting pores; (ii) triggering endoplasmic reticulum (ER) stress and unfolded protein response (UPR), thereby compromising proteostasis via dysfunction of the ubiquitin–proteasome system (UPS) and autophagy; (iii) impairing mitochondrial function and disrupting redox balance; (iv) interfering with endosomal–lysosomal as well as axonal and synaptic trafficking; and (v) activating stress‐kinase signaling and apoptotic pathways. In relation to therapeutic intervention, we review secretase‐targeting strategies, conformation‐selective antibodies, and their mixed clinical outcomes. An in‐depth understanding of the toxic action of pathogenic oligomeric species will be critical for translating these mechanistic insights into effective therapies that comprehensively target oligomer toxicity.

## 1 INTRODUCTION

Amyloidoses are a diverse group of diseases, encompassing more than 30 distinct types, all characterized by the misfolding and aggregation of specific proteins into insoluble, β‐sheet‐rich fibrils known as amyloids (DeToma et al., 2012; Gertz & Dispenzieri, 2020; Gurlo et al., 2010; Jeong & An, 2015; Kamatham et al., 2024; Knopman et al., 2021; Li & Liu, 2020; Nguyen et al., 2021; Rambaran & Serpell, 2008; Sekijima, 2015; Sipe et al., 2014; Tanner & Ostrem, 2024; Willbold et al., 2021; Zerr et al., 2024). In the past, it has been commonly proposed that amyloid fibrils primarily associate with toxicity. However, the connection between amyloid deposition and disease pathogenesis is complex, as amyloid formation is a multistep process that begins with protein misfolding, proceeds through the self‐association of these misfolded proteins in transient oligomeric intermediates, and ends in the accumulation of mature amyloid fibrils (Sinnige, 2022). In many cases, the presence of amyloid fibrils does not correlate well with the severity of clinical symptoms (Stefani, 2012), and it has been suggested that amyloid fibrils may act as inert sinks that sequester toxic precursor species (Bigi et al., 2022). Nevertheless, the limited correlation between fibril load and clinical severity does not imply that mature fibrils are uniformly benign. For example, once mature fibrils form, chaperone‐mediated disassembly can generate fragmented fibrils that nucleate further aggregation, suggesting that amyloid fibrils can contribute to protein misfolding under some conditions (Nachman et al., 2020; Tittelmeier et al., 2020). Additionally, injection of α‐synuclein fibrils into the striatum of mice causes the animals to manifest symptoms similar to those of Parkinson's disease in humans, suggesting that fibrils can also be neurotoxic (Luk et al., 2012). Increasing evidence supports conformation‐dependent behavior of amyloid fibrils (often referred to as strains/polymorphs), where distinct structural variants can differ in stability, seeding competence, spread, and biological impact (Lövestam et al., 2024; Schweighauser et al., 2020; Shi et al., 2021). Hence, fibrils may contribute to pathology through their structure‐dependent stability, polymorphism, persistence, and capacity to generate new seeds via fragmentation, whereas oligomers remain important as transient intermediates that can engage membranes and stress pathways early in the cascade. A comprehensive account of amyloid pathogenicity likely encompasses both soluble assemblies and filamentous forms, with their relative contributions varying across diseases, disease stages, and microenvironments.

In this review, however, we focus on the oligomeric species. At present, the amyloid oligomers have been recognized as cytotoxic species contributors across many protein misfolding disorders, including AD (associated with Aβ and tau), PD (linked to α‐synuclein), amyotrophic lateral sclerosis/frontotemporal dementia (ALS/FTD, involving misfolded TDP‐43, FUS, or SOD1), Huntington's disease (HD, associated with polyglutamine‐expanded huntingtin protein [polyQ htt]), T2DM (linked to IAPP and insulin), and systemic amyloidosis (linked to transthyretin [TTR] and immunoglobulin light chains [LC]) (Bargsted et al., 2017; Benilova et al., 2012; Caughey & Lansbury, 2003; Fang et al., 2014; Furukawa et al., 2006; Haass & Selkoe, 2007; Kayed et al., 2003; Lambert et al., 1998; Lasagna‐Reeves et al., 2011; Leitman et al., 2013; Maeda et al., 2007; Sangwan et al., 2017; Sengupta et al., 2016; Sikkink & Ramirez‐Alvarado, 2010; Sousa et al., 2001; Walsh & Selkoe, 2007; Winner et al., 2011; Zhao et al., 2009). Additionally, substantial evidence links soluble oligomeric intermediates to early cellular injury, including the formation of pore‐like assemblies in membrane that increase permeability, drive uncontrolled ion flux, and trigger Ca^2+^ dysregulation and downstream pathogenic cascades (Lashuel et al., 2002; Lee et al., 2017; Limbocker et al., 2019; Lin et al., 2001; Milardi et al., 2021; Mirzabekov et al., 1996; Quist et al., 2005; Sciacca et al., 2018). In vivo, the prion‐like behavior reported for oligomeric species further provides causal support, as intracerebral inoculation of preformed oligomers induces molecular and phenotypic changes that recapitulate key features of the corresponding proteinopathies (Clavaguera et al., 2009; Frost et al., 2009; Harper & Lansbury, 1997; Meyer‐Luehmann et al., 2006; Ren et al., 2009). Hence, defining structure‐toxicity relationships, gaining mechanistic insights about oligomer toxicity, and mapping the locations of distinct oligomer species within aggregation pathways remains a key knowledge gap and an area of intensive research.

This review summarizes recent advances in understanding the toxicity mechanisms of oligomeric species, as well as the roles of lipid composition, chaperone activity, oxidative stress, and cellular crowding in protein misfolding and aggregation. We also cover current therapeutic strategies that target protein aggregation and discuss potential reasons for their limited efficacy. Our goal is to review both current and new knowledge of protein misfolding and toxicity and to highlight existing gaps and potential targets for the development of therapies for amyloid‐associated diseases.

## 2 CELL MEMBRANE DISRUPTION

Oligomers of misfolded proteins, including Aβ (Bode et al., 2017; Kotler et al., 2015; Kotler & Ramamoorthy, 2018; Lin et al., 2001; Sahoo et al., 2020; Suzuki et al., 2013), α‐synuclein (Chen et al., 2015; Kayed et al., 2009; Lashuel et al., 2002), IAPP (Gurlo et al., 2010), PrP (Bahadi et al., 2003), and other amyloid‐forming proteins (Quist et al., 2005) interact with cellular membranes in an aberrant way. Depending on the subcellular localization of the precursor proteins, the plasma membrane, mitochondrial membrane, or organelle membranes may be affected (Camilleri et al., 2013; Gurlo et al., 2010; Mirzabekov et al., 1996).

### 2.1 Pore formation and membrane disruption

**FIGURE 1:**
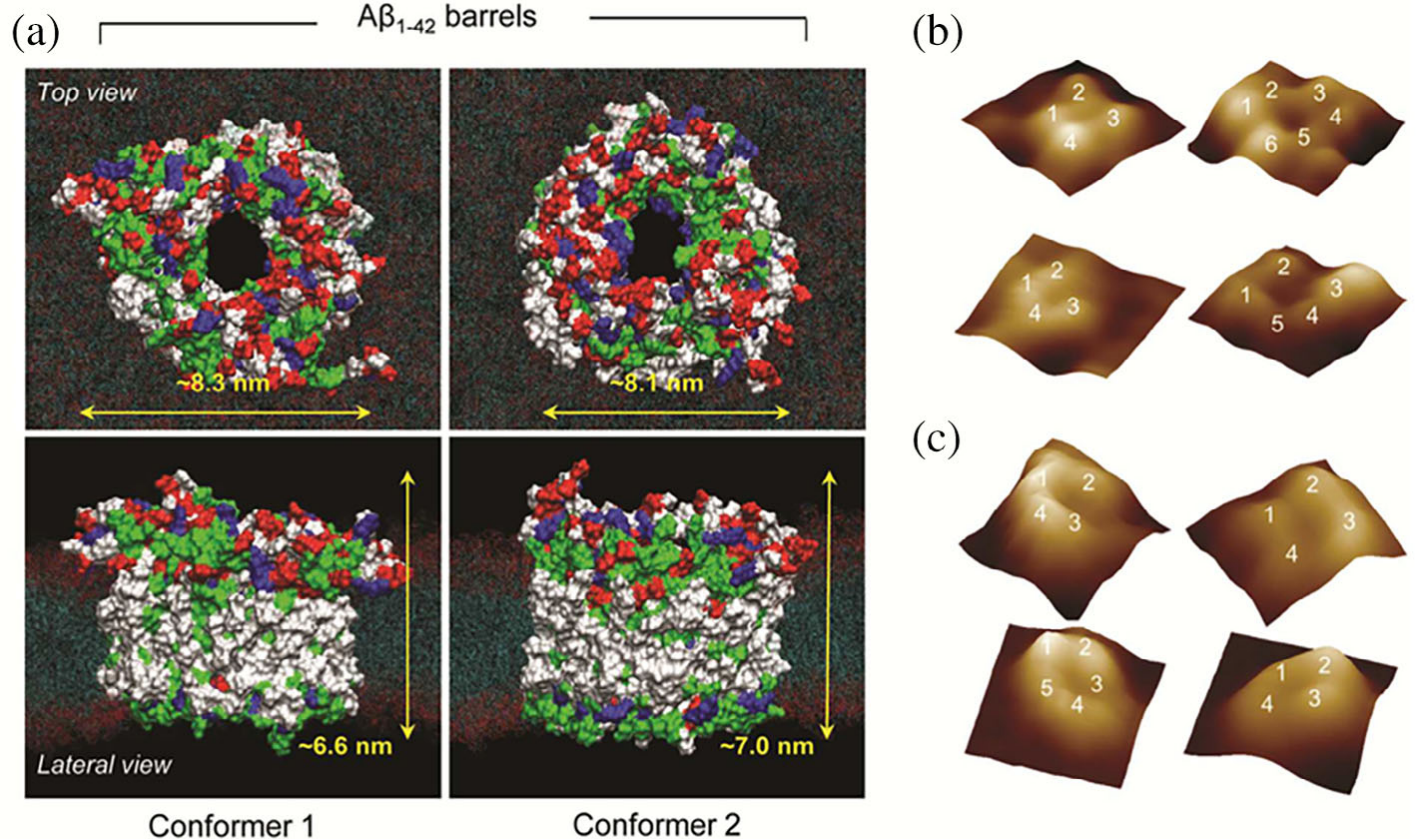
Top and lateral views of simulated (a) Aβ42 channel structures embedded in the DOPS/POPE lipid bilayer for the conformer 1 and 2 18‐mer barrels. The N‐terminus side is represented in the upper leaflet, and the turn region is represented in the lower leaflet. In the surface representation of the barrel, hydrophobic residues are shown in white, polar and Gly residues are shown in green, positively charged residues are shown in blue, and negatively charged residues are shown in red. For DOPS/POPE lipids, red dots denote the head groups, and cyan dots represent the lipid tails. Representative 3D AFM images of pore structures of Aβ42 in BTLE (b) and in DOPS/POPE (c) membranes (figure reproduced from Lee et al., 2017).

In some cases, misfolded proteins assemble into pore‐shaped oligomers, such as annular or β‐barrel‐like structures, within cellular membranes. Similar to non‐selective ion channels, these pores of amyloid proteins increase membrane permeability. This leads to uncontrolled ion flow and disrupts ionic balance within the cell (Lashuel et al., 2002; Lee et al., 2017; Limbocker et al., 2019; Lin et al., 2001; Mirzabekov et al., 1996; Quist et al., 2005; Sciacca et al., 2018). Electrophysiological recordings have directly confirmed ion conductance for pores formed by Aβ, α‐synuclein, and IAPP oligomers, while atomic force microscopy (AFM) and molecular dynamics (MD) simulations have provided complementary structural evidence for their pore‐like organization (Arce et al., 2011; Capone et al., 2009; Connelly et al., 2012; Jang et al., 2007; Lee et al., 2017; Mirzabekov et al., 1996; Quist et al., 2005) (see Figure 1). A two‐step mechanism has been proposed for membrane disruption. In this model, aggregation proceeds through initial pore formation, followed by detergent‐like fragmentation of the membrane (Brender et al., 2012; Sciacca et al., 2012).

The consequent dysregulation of calcium is a major contributor to the toxicity of the pore‐shaped oligomers. Unregulated Ca^2+^ entry through these pores impairs synaptic function and triggers pathogenic cascades such as the activation of enzymes like calpains and caspases (Foster, 2007; Lashuel et al., 2002; Quist et al., 2005). Mitochondria are particularly vulnerable to calcium overload, as excessive calcium can trigger the opening of the mitochondrial permeability transition pore, causing a collapse of its membrane potential and release of pro‐apoptotic factors (Caroppi et al., 2009; Di Scala et al., 2016; Lee et al., 2017). Calcium imaging and electrophysiological studies have shown that oligomer‐induced Ca^2+^ influx is not random but often displays pulsatile or ion channel‐like behavior, supporting the concept that amyloid oligomers form non‐selective, pore‐like ion channels (Capone et al., 2009; Connelly et al., 2012).

Amyloid oligomers can also disrupt lipid bilayers without forming discrete channel‐like structures. Studies have shown that soluble oligomers of Aβ, α‐synuclein, IAPP, and other amyloidogenic peptides can increase lipid bilayer conductance and compromise membrane integrity (Sciacca et al., 2021). The interaction of amyloid oligomers with lipid membranes can also result in structural defects and discontinuities. In the carpet model, oligomers adsorb to and coat the membrane surface, disrupting lipid packing and weakening the bilayer, increasing permeability and altering membrane potential (Tian et al., 2021; Walsh et al., 2014).

Lipid composition and concentration are strong modulators of binding and insertion depth of the oligomers (Azouz et al., 2019). Consistent with this, Kenyaga et al. proposed that lipid‐protein interactions actively remodel the bilayer, creating curved “edge/defect” regions and local membrane thinning as shown by NMR studies with neuron‐derived synaptic membranes (Kenyaga et al., 2022). The extent of this disruption is critically dependent on oligomer size. Smaller Aβ oligomers readily solubilize into membranes and are more toxic than the larger oligomers, which are lipid‐insoluble (Yang et al., 2017). This toxicity of smaller Aβ oligomers likely arises from their unique ability to alter membranes through pore formation and lipid extraction (Mrdenovic et al., 2019).

**FIGURE 2:**
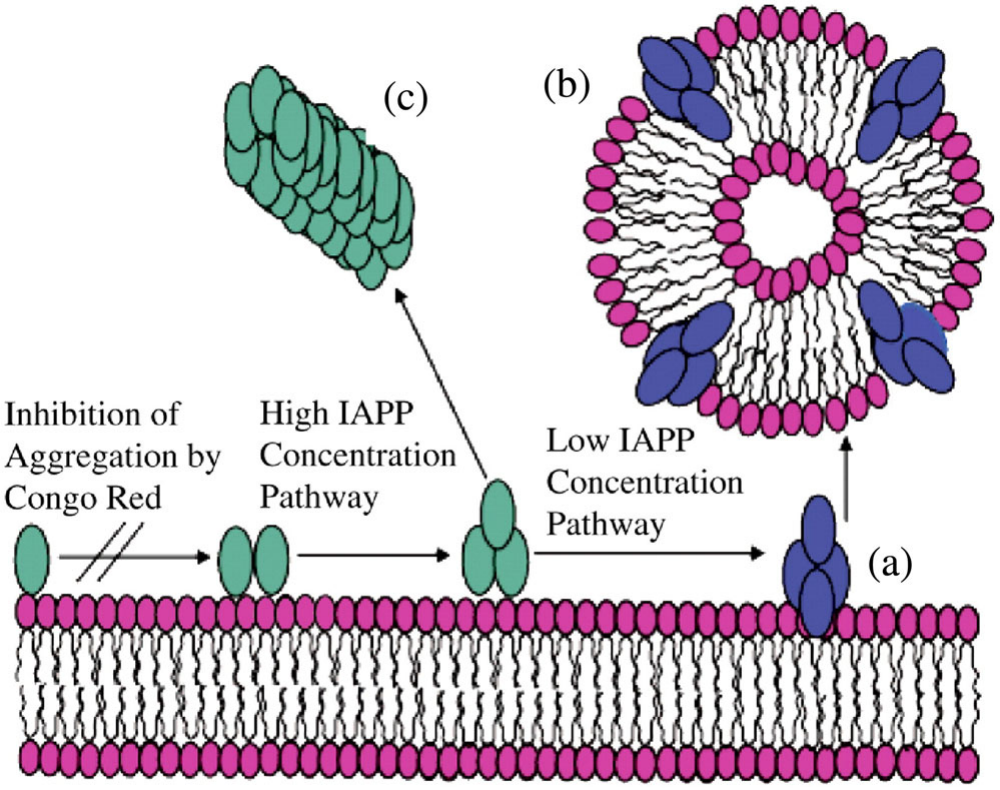
A schematic model for membrane fragmentation by human IAPP20‐29 based on solid‐state NMR studies on aligned lipid bilayers. (a) At lower concentrations, the peptide aggregates on the lipid bilayer surface to form an intermediate state that is capable of extracting phospholipid molecules from the bilayer and generating peptide‐lipid vesicles (b). At higher concentrations, fiber formation follows an alternate pathway bypassing the formation of this intermediate (c) (figure reproduced from Brender et al., 2007).

Like detergents, amyloid oligomers can sequester lipids from membranes. This lipid siphoning alters the composition, curvature, permeability, and fluidity of the membranes (Sciacca et al., 2018). For example, hIAPP and Aβ can form micelle‐like oligomers that solubilize membrane components (Bode et al., 2019; Brender et al., 2012; Sciacca et al., 2012). Figure 2 illustrates the detergent‐like mechanism of membrane disruption proposed for the human IAPP fragment, based on a solid‐state NMR study (Brender et al., 2007). In this model, amphipathic peptide segments insert into the bilayer, disrupt lipid packing, and extract lipids into peptide‐lipid complexes, increasing membrane permeability without forming stable pores.

## 3 INTERFERENCE WITH AXONAL TRANSPORT

Oligomers of tau, TDP‐43, and α‐synuclein sequester motor proteins like kinesin and dynein or destabilize microtubule networks. The result is axonal swelling, depletion of synaptic vesicles, and the accumulation of organelles and other essential cargo (De Vos et al., 2008; Millecamps & Julien, 2013). This altered axonal transport interferes with neuronal communication and survival (Kim et al., 2011; Sanchez‐Varo et al., 2012; Shah et al., 2009).

## 4 SYNAPTIC DYSFUNCTION

Aβ oligomers abnormally interact with and alter AMPA‐ and NMDA‐type glutamate receptors, which are central mediators of postsynaptic excitatory transmission (Guntupalli et al., 2016; Li et al., 2011; Shankar et al., 2008; Texidó et al., 2011; Tu et al., 2014). Perturbed NMDA/AMPA signaling is also associated with dysregulation of the Wnt/β‐catenin pathway, which is crucial for synapse formation and the maintenance of synaptic plasticity (Inestrosa et al., 2007; Parihar & Brewer, 2010).

Presynaptic function is also impaired by Aβ and α‐synuclein oligomers through their binding to the SNARE complex or its regulatory partners, which leads to defective vesicle docking, fusion, and recycling. These abnormal interactions ultimately deplete synaptic vesicle pools and reduce neurotransmitter release (Cai et al., 2023; Choi et al., 2013; Fagiani et al., 2021; Ovsepian et al., 2018; Tanaka et al., 2019; Yang et al., 2015). Because vesicle cycling is essential for the recycling of postsynaptic receptors, these presynaptic defects result in decreased surface expression of glutamatergic and GABAergic receptors, weakening excitatory and inhibitory signaling (Mele et al., 2019; Ulrich, 2015).

## 5 ENDOSOMAL AND LYSOSOMAL TRAFFICKING

Amyloid oligomers, including Aβ, α‐synuclein, and tau, also interfere with endosomal–lysosomal trafficking pathways (Lai et al., 2021; Marshall et al., 2020). Such interference leads to cargo accumulation, altered membrane dynamics, and reduced hydrolase activity, interfering with the degradation of misfolded proteins. Specifically, soluble Aβ oligomers accumulate within early endosomes, where they dysregulate endosomal maturation and fusion with lysosomes (Nixon, 2017; Park et al., 2022; Perez et al., 2015; Poon et al., 2013; Zhang et al., 2022). Similarly, α‐synuclein oligomers impair Rab5‐ and Rab7‐mediated vesicular trafficking (Volpicelli‐Daley et al., 2014), and tau oligomers disrupt endosomal sorting and lysosomal acidification (Lai et al., 2021). Collectively, these trafficking defects establish a feed‐forward cycle wherein a compromised proteostasis system accelerates the abnormal accumulation of proteinaceous deposits.

## 6 INDUCTION OF CELLULAR STRESS

### 6.1 Endoplasmic reticulum stress

The endoplasmic reticulum (ER) manages misfolded proteins by activating the unfolded protein response (UPR). While the UPR restores proteostasis by upregulating chaperones and clearance pathways, chronic UPR activation leads to sustained ER stress and apoptosis. Oligomers of Aβ and α‐synuclein have been shown to disrupt ER function and chronically activate the UPR, causing neuronal death in AD and PD models (Ajoolabady et al., 2022; Hasan et al., 2024). The elevated load of misfolded proteins in the ER can also convert surrounding proteins into aberrant forms, thereby accelerating their accumulation. The oligomeric species can also bypass conventional ER‐to‐Golgi trafficking and be secreted via unconventional secretory pathways, such as ER‐Golgi‐independent vesicular transport or direct plasma membrane translocation. The result is extracellular deposition and neuroinflammation (Borland & Vilhardt, 2017; Nickel & Rabouille, 2009). Consequently, mitigating ER stress is a possible strategy for the reduction of aggregation burden. For example, expression of huntingtin (htt) with abnormal polyglutamine (polyQ) expansions induces ER stress, and alleviating ER stress has been shown to decrease htt aggregation (Reijonen et al., 2008).

### 6.2 Oxidative stress and mitochondrial dysfunction

Amyloid oligomers trigger oxidative stress through multiple converging pathways. Oligomers promote reactive oxygen species (ROS) by binding redox‐active metals (Cu, Fe) at the cell surface, in endosomes, and in the cytosol (see Section 10.1). Another source of oxidative stress is oligomer‐induced hyperactivation of NMDA‐ and AMPA‐type glutamate receptors at synapses. The hyperactivated receptors drive excessive calcium into the cell, stressing mitochondria and increasing ROS production from the electron transport chain (De Felice et al., 2007; Talantova et al., 2013). In parallel, oligomers permeabilize membranes, including plasma, synaptic, ER/endolysosomal, and mitochondrial membranes, creating pores that drive pathological ion flux, which further amplifies oxidative stress (Butterfield & Halliwell, 2019) (discussed in Section 2).

The primary mechanism of oxidative stress is the direct interaction of oligomers formed by misfolded proteins, such as tau, Aβ, α‐synuclein, and SOD1 with mitochondria, which is the main source of ROS in many cell types including neurons (Cozzolino et al., 2009; Devi et al., 2008; Lim et al., 2010; Lindström et al., 2017; Reddy, 2006; Shafiei et al., 2017; Shirendeb et al., 2011; Umeda et al., 2011; Wang et al., 2009). Resulting from oligomer accumulation is the disruption of key mitochondrial processes, including fission/fusion dynamics, membrane potential, and electron transport chain (ETC) activity (Manczak et al., 2011; Pagani & Eckert, 2011; Pantiya et al., 2020).

Calcium overload is a central contributor to oligomer‐induced mitochondrial toxicity. Driven by oligomer‐membrane interactions, elevated Ca^2+^ influx triggers mPTP opening, causing mitochondrial depolarization and the initiation of apoptosis (Caroppi et al., 2009; Di Scala et al., 2016; Lee et al., 2017; Moreira et al., 2002). Abnormal levels of intracellular calcium activate phospholipases, proteases (such as calpains), and nitric oxide synthases, which also contribute to the generation of ROS (Görlach et al., 2015; Misrani et al., 2021). Furthermore, ROS progressively damages mitochondria by oxidizing major biomolecules, leading to protein misfolding, lipid peroxidation, and DNA damage. These injuries impair ATP synthesis and dysregulate signaling pathways that govern cell survival, differentiation, and repair, creating a feed‐forward cycle of mitochondrial dysfunction (Butterfield & Halliwell, 2019; Görlach et al., 2015; Hewitt & Degnan, 2022; Li et al., 2013; Lin & Beal, 2006; Zhou et al., 2022).

## 7 ALTERED PROTEIN DEGRADATION AND CLEARANCE

Misfolding and oligomerization of amyloid proteins significantly disrupt the ubiquitin‐proteasome system (UPS) and autophagy pathways, both of which are essential for maintaining proteostasis (Chisholm et al., 2022; McKinnon et al., 2020; Tydlacka et al., 2008; Urushitani et al., 2002). The oligomers can inhibit the UPS through various mechanisms. The proteasome is most efficient at processing single, unfolded proteins. However, as oligomer size increases, proteasomal processing becomes progressively impaired, ultimately leading to the cessation of degradation (Pohl & Dikic, 2019; Stefanis et al., 2001; Tanaka et al., 2001). Oligomers of neurodegenerative proteins, such as α‐synuclein, Aβ, and tau, can also initiate the aggregation of not only themselves but also seed the aggregation of one another and other cellular proteins, thereby increasing the overall burden of misfolded proteins (Bennett et al., 2005; Ivanova et al., 2021). Moreover, the fragments produced during proteasomal degradation of misfolded proteins can be more prone to forming toxic oligomers. For example, the digestion of α‐synuclein by the 20S proteasome subunit generates highly aggregative fragments, which accelerate misfolding and aggregation of full‐length protein (Liu et al., 2003).

UPS‐mediated clearance relies on ubiquitin tagging by the E1–E2–E3 enzyme cascade. When misfolded proteins oligomerize, the enzymatic docking sites can become buried, reducing enzyme access and ubiquitination. In huntingtin, phosphorylation at Ser421 signals for degradation. However, Ser421 phosphorylation of mutant huntingtin is reduced because oligomerization sterically blocks access to the phosphorylation sites. This impairment directly leads to poor targeting by UPS and subsequent clearance of huntingtin oligomers (Thompson et al., 2009). Oligomerization can also disrupt the pool of ubiquitin, which assembles into Lys‐linked chains to tag misfolded proteins (ubiquitination) for degradation. For example, expression of mutant FUS and TDP‐43 in neuronal‐like cells causes a redistribution of ubiquitin and depletion of the free ubiquitin pool. This disruption is associated with the formation of misfolded TDP‐43 and FUS inclusions, which in turn sequester ubiquitin and other proteostasis components (Farrawell et al., 2020; Riemenschneider et al., 2022; Tran & Lee, 2022). In some instances, such as inclusions of C9orf72 associated with ALS, the active recruitment of 20S proteasomal subunits within the aggregates directly disrupts proteasomal function (Guo et al., 2018). Hence, functional disruption of the UPS and autophagy by oligomers exacerbates the accumulation of misfolded proteins (e.g., α‐synuclein, huntingtin, TDP‐43) and activates cellular stress responses, ultimately leading to proteostasis failure (Filimonenko et al., 2007; Metcalf et al., 2012; Winslow et al., 2010).

There is substantial evidence indicating that autophagy is hindered by protein misfolding, a topic that has been extensively reviewed in the literature (Barmaki et al., 2023; Feng et al., 2020; Filimonenko et al., 2007; Metcalf et al., 2012; Winslow et al., 2010). The UPS and autophagy are highly interconnected, with misfolded proteins, oligomers, and inclusions often being processed by multiple degradation pathways concurrently (Li et al., 2022; Pohl & Dikic, 2019). The predominance of each pathway depends on the stage of aggregation, and in some cases, several pathways operate simultaneously to clear the toxic species (Stefanis et al., 2019). An example illustrating the complexity of protein degradation is α‐synuclein. The UPS typically degrades soluble, monomeric α‐synuclein. As α‐synuclein aggregates grow larger, autophagy, specifically macroautophagy, takes over the UPS (Ebrahimi‐Fakhari et al., 2011; Lee et al., 2004; Webb et al., 2003). Additionally, chaperone‐mediated autophagy (CMA), a lysosomal degradation route independent of macroautophagy, can also target α‐synuclein for clearance, despite the lack of a canonical degradation motif (Bayati & McPherson, 2024; Stefanis et al., 2019; Xilouri et al., 2013). Furthermore, calpains and metalloproteases can proteolytically degrade α‐synuclein, providing an alternative clearance pathway (Xilouri et al., 2013). Misfolded α‐synuclein itself impairs both UPS and autophagy, creating a positive feedback loop in which aggregate accumulation further compromises clearance (McKinnon et al., 2020; Winslow et al., 2010). This interdependence among degradation pathways complicates efforts to define the precise mechanisms of oligomer clearance, and the results often depend on the specific experimental model used.

In summary, both UPS and autophagy are disrupted by misfolding, with the extent and mechanism of impairment depending on the specific protein involved and the stage of aggregation. The failure to clear misfolded proteins triggers cellular stress responses, activating the unfolded protein response (UPR) and ER stress pathways (discussed in Section 6.1 and 8). These stress responses further increase the protein burden, leading to inflammation, mitochondrial dysfunction, and ultimately, cell death.

In the case of TDP‐43 and FUS misfolding which are RNA‐binding proteins (RNBs), the toxicity can also be tightly intertwined with RNA metabolism (splicing, transport, surveillance/decay) (Da Cruz & Cleveland, 2011; Rummens & Da Cruz, 2025; Zaepfel & Rothstein, 2021). Therefore, interventions aimed at restoring RNA‐processing capacity should be as important as those that focus on preventing aggregate formation. In support of RNA‐based toxicity, neuronal model studies show that augmenting RNA surveillance via UPF1 can mitigate TDP‐43– and FUS‐associated toxicity, supporting RNA‐processing pathways as actionable therapeutic entry points (Barmada et al., 2015).

In addition to cell‐intrinsic proteostasis pathways, Aβ is cleared from the brain through blood–brain barrier (BBB) transport and perivascular drainage. Under physiological conditions, Aβ levels are commonly regulated by low‐density lipoprotein receptor‐related protein 1 (LRP1) and advanced glycation end products (RAGE) receptor, both expressed by BBB endothelial cells. LRP1 mediates Aβ transport from the brain to the blood for peripheral disposal, while the RAGE receptor binds circulating Aβ and facilitates its entry into the brain. Studies have shown that decreased LRP1 and increased RAGE activity are associated with neurodegeneration (Greenberg et al., 2020; Petrushanko et al., 2023; Wang et al., 2021). Such defective clearance due to vascular Aβ deposition is observed in AD and in cerebral amyloid angiopathy (CAA) (Gireud‐Goss et al., 2021; Petrushanko et al., 2023; van Veluw et al., 2024).

A possible mechanism of aggregation is that the impaired vascular clearance promotes a rise in local Aβ levels, beyond a critical threshold favorable for the Aβ self‐association into oligomers and fibrils (Goto et al., 2024; Nguyen et al., 2021), that deposit on vessel walls, hence altering BBB function (Chen et al., 2025; Petrushanko et al., 2023; Tang et al., 2025). Because oligomers are transient and heterogeneous, their detection and characterization pose challenges, leaving uncertainty about which Aβ assemblies drive specific pathological outcomes (see the accompanying review for strategies to study oligomers). Because impaired perivascular clearance is considered a major driver of vascular Aβ accumulation in CAA, strategies that enhance drainage and vascular clearance have been proposed as potential therapeutic approaches (Bonnar et al., 2025; van Veluw et al., 2024).

## 8 APOPTOTIC PATHWAYS

Evidence suggests that amyloid oligomers activate canonical pathways of apoptosis. In the extrinsic (death‐receptor) pathway, Aβ aggregates promote caspase‐8 activation by engaging or upregulating neuronal death receptors, Fas, TNFR1, and the TRAIL receptors DR4/DR5, thereby recruiting adaptor proteins and assembling the DISC (Anzovino et al., 2023; Fossati et al., 2012; Li et al., 2004; Su et al., 2003). Activated caspase‐8 can either directly activate caspase‐3 or initiate mitochondrial damage by cleaving Bid, a pro‐apoptotic Bcl‐2 family protein that amplifies intrinsic signaling (Folin et al., 2006; Ivins et al., 1999). In the intrinsic (mitochondrial) pathway, Aβ binding activates BAX/BAK, which leads to cytochrome c release, apoptosome assembly, and caspase‐9 activation (Kim et al., 2014; Picone et al., 2009).

Oligomers also activate noncanonical pathways involving stress‐activated kinases such as p38 MAPK and c‐Jun N‐terminal kinase (JNK), which act upstream of caspases. Among the earliest responses to Aβ oligomers exposure is increased activation of JNK and p38 mitogen‐activated protein kinase (p38 MAPK) in both in vitro and in vivo models of AD (Morishima et al., 2001; Munoz & Ammit, 2010; Sun et al., 2003). Both JNK and p38 MAPK are members of the MAPK family, which responds to oxidative stress, protein misfolding, and DNA damage (Kyriakis & Avruch, 2012).

Pharmacological or genetic inhibition of JNK alleviates neuronal apoptosis induced by Aβ oligomers, suggesting that JNK mediates downstream toxicity (Awasthi et al., 2005; Yao et al., 2005). Mechanistically, JNK phosphorylates pro‐apoptotic Bcl‐2 family proteins, promoting mitochondrial outer membrane permeabilization, which enhances apoptosis through cytochrome c release, apoptosome assembly, and activation of initiator caspase‐9, followed by executioner caspase‐3 (Mattson, 2000; Resende et al., 2008). In parallel, activated by Aβ oligomers, p38 MAPK promotes inflammatory signaling through NF‐κB and AP‐1 pathways, enhancing microglial activation and facilitating the release of pro‐inflammatory cytokines (Canovas & Nebreda, 2021; Munoz & Ammit, 2010; Robinson & Cobb, 1997). p38 MAPK also phosphorylates tau, linking the toxicity of Aβ oligomers to tau pathology (Maphis et al., 2016). Importantly, activation of the MAPK family is not restricted to Aβ pathology. α‐Synuclein aggregation has also been linked to the JNK cascade, contributing to cortical neuronal death (Ferrer et al., 2001; Suzuki et al., 2024), and both JNK and p38 MAPK have been implicated in toxicity associated with misfolded FUS, TDP‐43, and polyQ htt (Gogia et al., 2020; Liu, 1998; Perrin et al., 2009; Reijonen et al., 2010; Sama et al., 2017; Zhan et al., 2015). Hence, the aberrant activation of JNK and p38 MAPK by misfolded proteins establishes a cycle of oxidative stress, inflammation, and apoptosis.

As discussed in the previous sections, oligomers trigger upstream stressors, including Ca^2+^ overload, ER stress, lysosomal leakage, and ROS. In turn, these stressors activate the extrinsic (caspase‐8) and/or intrinsic mitochondrial (caspase‐9) pathways, converging on executioner caspases‐3/7, or activate caspase‐3 even in the absence of cytochrome c release (Yapryntseva et al., 2024). These apoptotic mechanisms are context‐dependent and vary with cell type, oligomer species, and disease stage. Thus, defining the dominant pathways under specific conditions is essential for the rational design of effective strategies to counter oligomer‐induced cell death.

## 9 NEUROINFLAMMATION AND MICROGLIAL RESPONSES

Microglial activation and neuroinflammation are important downstream responses to protein misfolding and aggregation, with microglia functions varying across disease stages. In the early stages, microglia can exert protective effects by promoting the uptake and clearance of extracellular aggregates. However, with aging and chronic proteotoxic stress, microglial phagocytosis can become dysregulated. This dysregulation is associated with reduced clearance of Aβ aggregates and can contribute to synaptic dysfunction as the disease progresses (Brown et al., 2026; Heneka et al., 2025; Heppner et al., 2015; Hickman et al., 2018; Webers et al., 2020).

## 10 COFACTORS AND THE CELLULAR ENVIRONMENT

### 10.1 Metal ions

**FIGURE 3:**
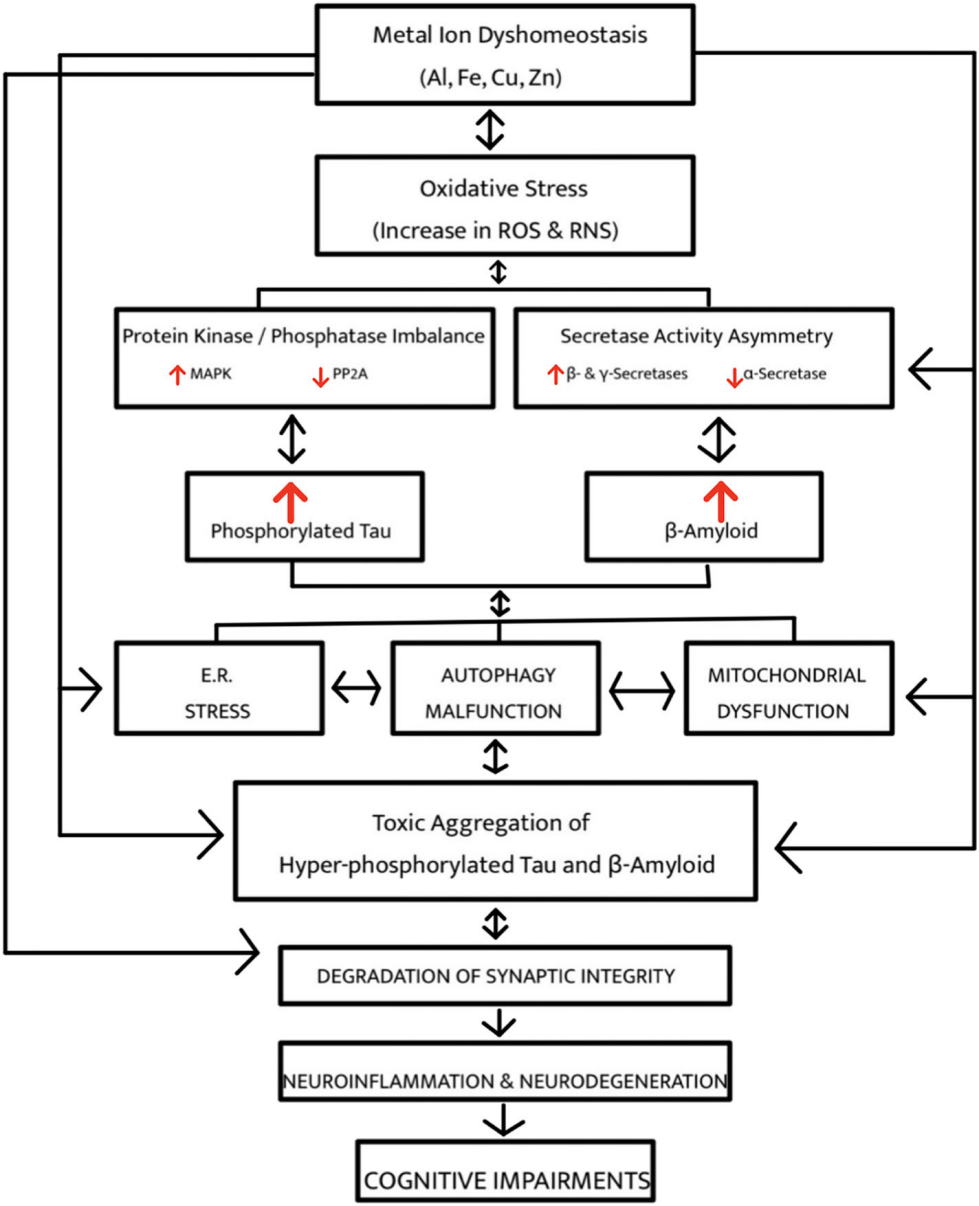
This schematic demonstrates the multi‐level effects of metal ion imbalances on the multifarious toxicities associated with AD pathology, including oxidative stress, Aβ and Tau accumulation, neuroinflammatory responses, etc., leading to downstream cognitive dysfunction (figure reproduced from Das et al., 2021).

Redox‐active metal ions like copper, zinc, and iron play a critical role in the pathology of amyloidogenic proteins such as α‐synuclein, Aβ, and amylin (Binolfi et al., 2006; Miller et al., 2012; Sinopoli et al., 2014; Yi & Lim, 2023), see Figure 3. Metal interactions can stabilize β‐sheet–rich aggregation and aid membrane interactions. For example, copper coordination by Aβ increases its positive charge, enhancing its affinity for negatively charged membranes (Smith et al., 2006; Watt et al., 2012). The membrane‐bound oligomers concentrate redox‐active species, such as copper and iron, near membrane lipids, which initiates lipid peroxidation and bilayer disruption (Yi & Lim, 2023). Furthermore, through the same chelation mechanism within intracellular compartments, oligomers catalyze Fenton/Haber‐Weiss reactions. This produces highly reactive hydroxyl radicals that oxidize and cross‐link proteins (Das et al., 2021). Consistent with in vitro evidence that metals promote amyloid oligomerization, postmortem analysis of AD brains shows metal‐ion dyshomeostasis and miscompartmentalization (Gaggelli et al., 2006; Scott & Orvig, 2009). Furthermore, the accumulation of metals in amyloid plaques is higher than in surrounding tissue (Bush & Tanzi, 2008; Lovell et al., 1998; Wang et al., 2018). Because metal ions are directly involved in protein misfolding, targeting metal‐amyloid interactions by metal chelators and metal‐protein‐attenuating compounds is currently an actively pursued therapeutic strategy (Choi et al., 2010; Savelieff et al., 2014; Singh et al., 2024).

### 10.2 Lipids

Cholesterol‐rich and ganglioside‐containing lipid microdomains (lipid rafts) are strongly linked to amyloid protein misfolding and oligomerization of Aβ, α‐synuclein, IAPP, and prion protein (Christensen et al., 2021; Fanning et al., 2020; Kumar et al., 2023a; Kumar et al., 2023b; Matsuzaki, 2020; Mirdha, 2024; Pantelopulos et al., 2024; Walsh et al., 2014; Zhaliazka et al., 2023). These microdomains are characterized by tightly packed acyl chains and reduced membrane fluidity, which promote abnormal amyloid–lipid interactions. For example, cholesterol interacts with the hydrophobic region of IAPP, stabilizing “insertion‐ready” conformations that disrupt lipid packing once inserted into the membrane (Sciacca et al., 2016). In neuronal membranes, α‐synuclein preferentially forms aggregates where cholesterol‐ and sphingolipid‐rich rafts are located (Fanning et al., 2020; Fortin et al., 2004). Similarly, cholesterol‐rich domains facilitate Aβ oligomerization into pore‐like structures that are permeable to calcium (Di Scala et al., 2014). Consistent with these findings, inhibition of cholesterol synthesis reduces Aβ aggregation in some cellular models (Wakabayashi & Matsuzaki, 2007). The effect of cholesterol synthesis in the case of α‐synuclein is more complex. Studies have shown that significant cholesterol reduction destabilizes lysosomal membranes and increases their permeabilization (Eriksson et al., 2017). This duality of cholesterol is in line with epidemiological findings, which associate high and low cholesterol levels with increased risk of PD (De Lau et al., 2006; Paul et al., 2015).

Cholesterol‐rich lipid rafts not only promote amyloid aggregation but also act as platforms that cluster receptors (e.g., TLR4). This clustering triggers pro‐inflammatory signaling and JNK upregulation, pathways linked to cell death (Miller et al., 2020; Tall & Yvan‐Charvet, 2015). Consequently, the inflammatory toxicity resulting from stabilized cholesterol rafts can be due to not only direct oligomer binding but also other cellular mechanisms, as demonstrated for Aβ and α‐synuclein (Ding et al., 2025; Fortin et al., 2004).

Similar to cholesterol, gangliosides, specifically GM1, accelerate the aggregation of both Aβ and α‐synuclein. Aβ preferentially binds GM1 over zwitterionic lipids (PC, SM) or other anionic lipids (PS, PG), primarily through electrostatic interactions between positively charged residues and the sialic acid group of GM1 (Matsuzaki, 2020; McLaurin & Chakrabartty, 1996). Both α‐synuclein and Aβ share a common GM1‐binding motif, which is 12 residues long (residues 5–16 in Aβ and 34–45 in α‐synuclein). This region has been targeted in the design of inhibitors to block lipid binding and aggregation (Yahi & Fantini, 2014).

Gangliosides can have dual, concentration‐dependent effects on amyloid aggregation. In the case of IAPP, high ganglioside‐to‐peptide ratios reduce aggregation, while low ratios promote clustering of peptides on limited GM1 sites, facilitating aggregation (McCalpin et al., 2024). Additionally, lipids modulate one another by tuning membrane phase behavior: cholesterol promotes GM1 clustering into ordered raft‐like domains that serve as amyloid‐binding/nucleation sites. For example, Aβ40 aggregates readily on GM1/SM/cholesterol mixtures, whereas in GM1/PC‐rich liquid‐crystalline liposomes, Aβ40 monomers remain more uniformly dispersed and less prone to aggregation (Kakio et al., 2001; Matsuzaki, 2020).

### 10.3 Molecular chaperones

Chaperones act at all stages of protein quality control: folding nascent chains or refolding misfolded chains (foldase activity), binding misfolded proteins to prevent aggregation (holdase activity), and disassembling aggregates (disaggregase activity) (Hipp & Hartl, 2024). Impairment at any stage can promote the accumulation of misfolded proteins and aggregates, and the chaperone involvement varies with disease stage and the specific protein involved.

At early stages of misfolding, chaperones reduce aggregation by stabilizing native conformers or clearing misfolded species. Clusterin, Hsp70, Hsp90, and BRICHOS domain–containing proteins either stabilize native states or promote degradation via the ubiquitin–proteasome system (UPS) and autophagy (Arispe & De Maio, 2018; Kettern et al., 2010; Mannini & Chiti, 2017; Rivera et al., 2018). GroEL, the bacterial homolog of Hsp60, which has holdase‐like activity, can delay Aβ42 fibrillization and prevent neuronal damage (Wälti et al., 2018). At advanced stages of misfolding, once mature fibrils have formed, the chaperones Hsp70, Hsp40, and Hsp110 act synergistically to disassemble fibrils of tau, α‐synuclein, and huntingtin (Montresor et al., 2025; Nachman et al., 2020; Schneider et al., 2021; Scior et al., 2018; Shorter, 2011). This disassembly activity results in fragmented fibrils, which can nucleate further aggregation (Nachman et al., 2020; Tittelmeier et al., 2020). To prevent the avalanche of misfolding that may result from fibril seeding, disassembly activity is coupled to UPS‐mediated degradation, as shown for VCP (Cliffe et al., 2019; Sontag et al., 2017). Under proteostatic overload, chaperones may sequester soluble oligomers into larger aggregates, reducing acute oligomer toxicity (Arrasate et al., 2004; Mannini et al., 2012; Walther et al., 2015). The accumulation of large aggregates introduces new stresses, such as impaired proteostasis, macromolecular crowding, and disruption of cellular compartments (Bäuerlein et al., 2017).

The experimental activation of selected chaperones has improved phenotypes and reduced aggregation in vivo and has been proposed as a therapeutic strategy. However, the highly complex and interdependent nature of the proteostasis network makes sustained chaperone activation difficult to maintain over prolonged times (Hipp & Hartl, 2024). Chaperones function in coordination with co‐chaperones, degradation pathways (UPS and autophagy), and stress response systems. Hence, altering chaperone expression levels can lead to compensatory imbalances. Because of this interconnectivity, chaperone‐based therapeutic approaches must be carefully considered to avoid disruptions to the proteostasis network as a whole.

## 11 PRION‐LIKE PROPERTY OF OLIGOMERIC SPECIES

Numerous studies have provided evidence that aberrant aggregates of Aβ, tau, α‐synuclein, TDP‐43, SOD1, and huntingtin can template their pathogenic conformation onto native proteins in a similar way to prion proteins (PrP^Sc^) (Clavaguera et al., 2009; Frost et al., 2009; Goedert et al., 2017; Harper & Lansbury, 1997; Meyer‐Luehmann et al., 2006; Ren et al., 2009). This prion‐like behavior can involve oligomers, protofibrils, or fibrillar fragments. Specifically, studies have shown that prion‐like oligomers are highly pathogenic: even at very low concentrations, they seed new toxic aggregates, transmitting the misfolded state within and between cells.

The prion‐like behavior provides direct causal evidence linking amyloid oligomers to toxicity. Intracerebral inoculation of preformed oligomers in mouse models induces molecular and phenotypic changes that recapitulate the pathology of the corresponding proteinopathy. For example, injection of tau oligomers causes synaptic dysfunction and mitochondrial impairment consistent with tauopathy‐related neurodegeneration (Lasagna‐Reeves et al., 2011). Similarly, Aβ oligomers trigger synaptic loss, neuroinflammation, insulin resistance, and exacerbation of tau pathology, as observed in AD (Cline et al., 2018; Cline et al., 2019). Injection of α‐synuclein oligomers produces dopaminergic neuron loss in the substantia nigra, as in PD pathology (Froula et al., 2019; Luk et al., 2012). Evidence suggests that cell‐to‐cell transmission of misfolded oligomers may be mediated by exosomal transport, vesicular trafficking, or tunneling nanotubes (Chakraborty et al., 2023; Fevrier et al., 2004; Frost et al., 2009).

Given their pathogenicity, the release and uptake of toxic oligomers are under intensive study as potential therapeutic targets for limiting the spread of pathogenic aggregation. Prion‐like oligomers are active at very low concentrations, which are often below the detection limits of conventional assays. Moreover, the cell‐to‐cell transfer can be reliably probed only in specialized cultures or organotypic models that preserve an in vivo‐like spatial organization and connectivity. Hence, the mechanistic basis of oligomer transmission remains largely undefined.

## 12 THE THERAPEUTIC CHALLENGE

### 12.1 Secretase‐targeting therapies

A major strategy in AD therapeutic research has been the secretases, proteases that cleave APP to generate Aβ. α‐, β‐, and γ‐secretases are the three main enzymes, which can process APP either into non‐toxic fragments or into aggregation‐prone Aβ peptides, and each enzyme has been a subject of drug development (Aisen, 2023; Egan et al., 2018; Hur, 2022; Kumar et al., 2018; Moussa, 2017; Neumann et al., 2018; Watkins & Vassar, 2024). The α‐secretase pathway, mediated by metalloproteases ADAM10/17, prevents Aβ generation by cleaving APP to release neuroprotective sAPPα (Hampel et al., 2021). However, other critical cellular processes also depend on this pathway, making it challenging to therapeutically activate this pathway. Conversely, β‐secretase (BACE1) initiates Aβ production. Studies have shown that inhibition of BACE1 reduces the initial cleavage of APP in a gene knockout mouse model, resulting in reduced Aβ production (Gabriele et al., 2022; Senechal et al., 2008). BACE1 inhibitors failed clinical trials, likely due to adverse effects, as BACE1 not only acts on APP but also cleaves multiple substrates essential for synaptic function and neuronal development (Hemming et al., 2009). Similarly, inhibition of γ‐secretase, an enzyme that cleaves APP to release aggregation‐prone Aβ42, has been unsuccessful. Although inhibitors, such as semagacestat (Eli Lilly) and avagacestat (Bristol‐Myers Squibb), reduced Aβ levels, these compounds produced severe adverse effects, which are explained by off‐target inhibition of Notch processing, essential for cell differentiation and tissue homeostasis (De Strooper, 2014; Vaz & Silvestre, 2020; Wilcock et al., 2008).

### 12.2 Small anti‐aggregation molecules

Another therapeutic approach involves the use of synthetic small molecules, often referred to as molecular chaperones, which can stabilize monomers and disrupt protein–protein interactions, driving fibrillization. A different class of compounds destabilizes mature fibrils, promoting their disassembly into smaller non‐fibrillar species that can be more readily cleared (Atanasova, 2025; Belluti et al., 2013; Kim et al., 2020; Sciacca et al., 2017; Yao et al., 2022). A potential drawback of disaggregating compounds, however, is that fibril destabilization may transiently increase the pool of soluble toxic oligomeric species. Among the molecules that progressed to clinical trials was tramiprosate (Alzhemed), a glycosaminoglycan mimetic that inhibited Aβ aggregation in vitro. However, Alzhemed was ineffective in improving cognition in Phase III trials (Manzano et al., 2020). A different compound, anapsos, a plant‐derived extract with anti‐amyloidogenic properties, showed no conclusive outcome in large‐scale studies (Alvarez et al., 2012). Natural polyphenols, such as curcumin, that inhibit Aβ aggregation have attracted much attention due to promising results in vitro and animal models. However, limited bioavailability and restricted CNS penetration of polyphenols in humans may help explain the inconclusive results of clinical trials (Shao et al., 2023). Similar to secretase inhibitors, a major limitation of the aggregation inhibitors is their poor specificity, suboptimal pharmacokinetics, low BBB permeability, and metabolic stability. Hence, current efforts focus on developing compounds with greater selectivity and improved delivery properties.

### 12.3 Immunotherapies (monoclonal antibodies)

Both active and passive immunization have been considered as immunotherapeutic strategies. Active immunization stimulates the patient's immune system to produce antibodies against the administered small fragments of the Aβ peptide. The post‐mortem analyses revealed significant reductions in amyloid plaque burden (Gilman et al., 2005; Wisniewski & Konietzko, 2008). However, the first clinical trial of this strategy, AN‐1792, was halted due to adverse inflammatory responses, including aseptic meningoencephalitis in a subset of patients.

Currently, passive immunization is the predominant strategy for delivering anti‐Aβ antibodies (Ladiwala et al., 2012; Qin et al., 2024; Usman et al., 2021; Zhang et al., 2024). At present, the FDA has approved aducanumab, lecanemab, and donanemab for the treatment of early‐stage AD. Aducanumab (Aduhelm), which preferentially binds aggregated Aβ (including oligomers and fibrils), produced mixed trial outcomes with modest cognitive benefit in some analyses (Budd Haeberlein et al., 2022; Mullard, 2021; Rahman et al., 2023) and was approved by the FDA but not by the European Medicines Agency. Lecanemab (Leqembi) and donanemab, both of which target plaque‐associated species, show robust amyloid reduction and delayed cognitive decline in clinical studies (Sims et al., 2023; Van Dyck et al., 2023). However, all three antibodies are associated with adverse vascular events, including cerebral edema and microhemorrhages.

Beyond clinical outcomes, biophysical studies provided insight into why antibodies that all “target Aβ” can behave very differently (Linse et al., 2020). Kinetic studies (often using ThT fluorescence) have suggested that conformational antibodies can preferentially modulate distinct microscopic steps in aggregate formation (e.g., monomer binding versus fibril‐surface–mediated processes). This leads to different effects on oligomer flux and fibril amplification. In particular, mechanistic “fingerprinting” suggests that aducanumab can substantially reduce the rate of oligomer formation compared to other clinical‐stage antibodies, consistent with selective interference in the aggregate formation (Linse et al., 2020). Complementary work has shown that inhibition of distinct microscopic steps can yield similar fibril readouts while producing very different oligomer populations (and therefore potentially different toxicity) (Aprile et al., 2017). Accordingly, because secondary nucleation is considered a major source of toxic oligomers in Aβ aggregation, it has been argued that targeting secondary nucleation can reduce oligomer generation more directly, whereas primary‐nucleation inhibition may primarily delay their appearance. In contrast, primary‐nucleation inhibition mainly delays the appearance of oligomers (Linse, 2019). These structure–activity and kinetic analyses support the notion that clinically used antibodies recognize distinct Aβ conformations/epitopes (including protofibril‐ or plaque‐enriched species), which likely contribute to both efficacy and side effects (Arndt et al., 2018; Johannesson et al., 2024; Söderberg et al., 2024). High‐resolution studies are beginning to resolve how antibody binding perturbs aggregate structure and dynamics. As shown for aducanumab, the antibody binding alters the dynamics of the N‐terminal region of Aβ_1–42_ fibrils while largely preserving the fibril core, providing a molecular view of the selective antibody engagement with amyloid aggregates (Palani et al., 2025).

There remains substantial room for therapeutic development. AD is a complex disorder, which is characterized not only by the pathogenic accumulation of Aβ, but also by tau pathology, neuroinflammation, oxidative stress, and metabolic dysfunction, processes likely contributing to limited efficacy of monoclonal antibody‐based immunotherapies that Aβ aggregation. As revealed by the kinetic studies, the timing of intervention (e.g., the stage of aggregation) is also critical and needs to be considered when determining at which step the antibodies act. Additionally, europathological changes begin decades before symptoms, and by the time of diagnosis, extensive neuronal injury may already be present, processes not limited to the amyloid burden. Moreover, early therapeutic effects may be difficult to detect because many trials rely on cognitive and functional endpoints that are relatively insensitive to molecular‐level changes. Additionally, poor blood–brain barrier penetration, patient‐to‐patient heterogeneity in disease mechanisms, and the difficulty of defining reliable clinical endpoints can also contribute to reduced antibody efficacy. Future progress will likely depend on combination therapies that address multiple disease pathways, supported by improved biomarker‐guided patient stratification and earlier preclinical detection (Kwon et al., 2020; Selkoe & Hardy, 2016).

### 12.4 Phospholipids as modulators of amyloid toxicity

IAPP, Aβ, and α‐synuclein exhibit a shared bilayer‐insertion and on‐membrane aggregation mechanism (Section 2, 5) (Brender et al., 2012; Korshavn et al., 2017; Kotler et al., 2014; La Rosa et al., 2016; Linse et al., 2020; Sciacca et al., 2018; Sciacca et al., 2020; Tempra et al., 2022), supporting the lipid‐chaperone hypothesis (LCH) that lipid interactions drive pathogenic assembly (Cohen et al., 2022; Sokolowski & Mandell, 2011). Targeting oligomer–lipid interactions holds broad therapeutic potential, particularly at early stages of aggregation, because many amyloid proteins exploit this interface. In designing therapy, it has to be taken into consideration that cellular membranes are heterogeneous and organized into dynamic, interdependent microdomains. Hence, it becomes difficult to selectively disrupt pathological lipid–protein interactions without interfering with essential membrane functions.

## 13 CONCLUSION AND FUTURE DIRECTIONS

Amyloid oligomers have been linked to the pathogenicity in many protein misfolding diseases, including AD, PD, T2DM, HD, and ALS/FTD. This review covers the complex mechanisms of oligomer‐induced toxicity, including current findings, unresolved questions, and directions for developing effective therapies.

**FIGURE 4:**
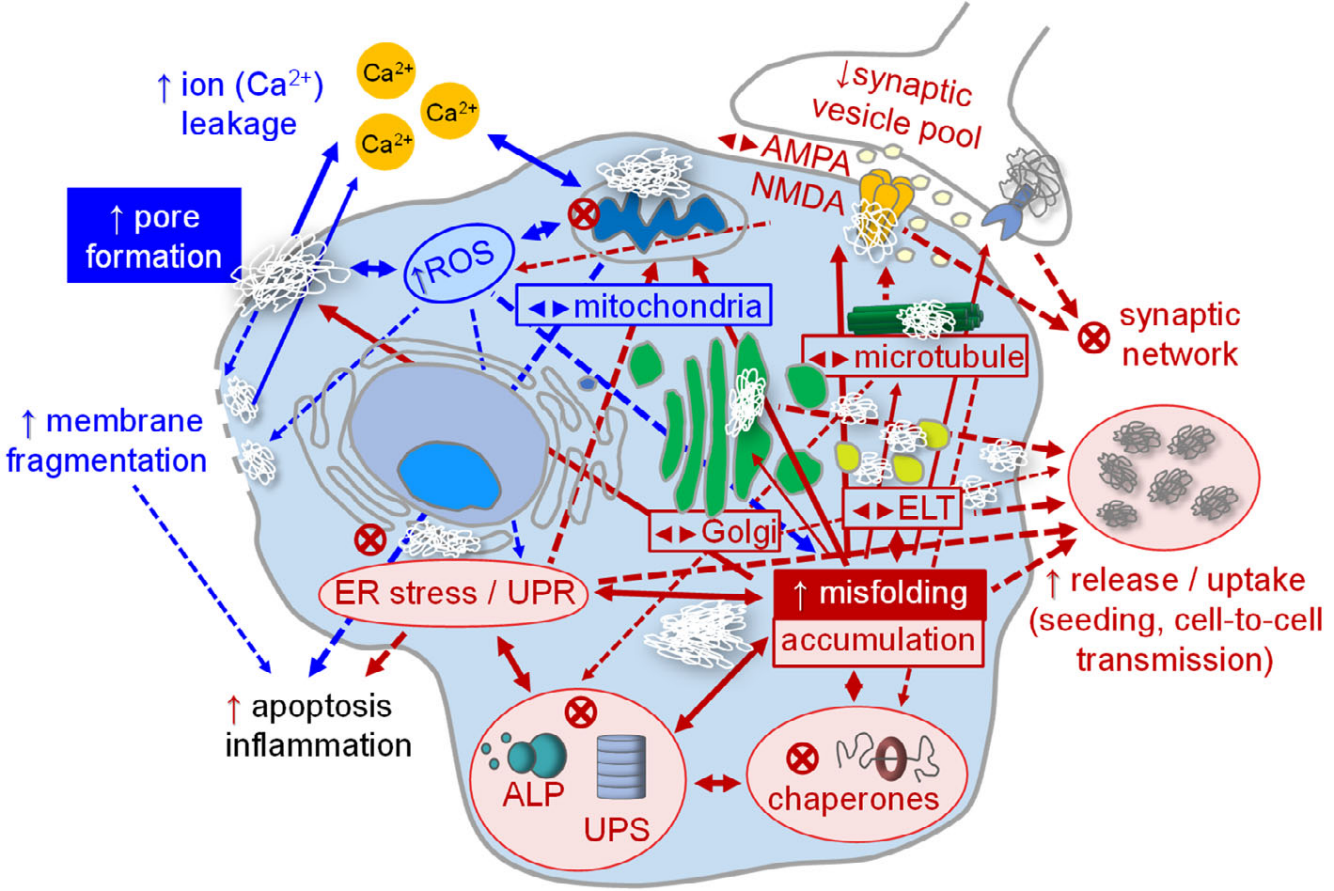
Schematic summarizing how diverse oligomer toxicity mechanisms connect through shared nodes and feed‐forward loops. Biophysical effects are shown in blue. Cellular processes associated with protein misfolding and oligomer formation are highlighted in red. Oligomers also impair microtubule/transport processes, contributing to synaptic vesicle depletion, altered synaptic receptor function, and breakdown of the synaptic network. Release/uptake pathways, such as vesicle‐mediated routes, that enable seeding and cell‐to‐cell transmission are also shown. The direction of the arrows denotes cause → effect; upper arrows ↑ indicate increased activity, dashed arrows indicate downstream connections; symbols indicate impaired/blocked processes. Thicker arrows highlight processes as higher‐weight/central links based on the body of supporting evidence and their role as convergence points in the toxicity cascade. ALP, autophagy‐lysosomal pathway; ELT, endosomal–lysosomal trafficking.

Protein misfolding and oligomer formation trigger many distinct mechanisms that contribute to cellular toxicity. Instead of viewing them separately, the different pathways, spanning membrane/pore (including lipid‐centered hypotheses), receptor/synapse signaling, proteostasis/ER stress, and mitochondria/ROS pathways can be viewed as distinct entry points into a shared network. Many of these toxicity pathways converge in shared stress nodes (oxidative stress/ROS overload and proteostasis failure) and common terminal outcomes (apoptosis, inflammation, and synaptic dysfunction). Figure 4 shows the connectivity between different toxicity pathways and marks the main stress nodes.

Although the dominant mechanisms are dependent on the context (protein, oligomer size/conformation, subcellular localization, membrane composition, and disease stage), a common initiating event is aberrant oligomer–membrane interaction (Arce et al., 2011; Capone et al., 2009; Connelly et al., 2012; Jang et al., 2007; Lee et al., 2017; Mirzabekov et al., 1996; Quist et al., 2005). The destabilized membrane integrity due to amyloid oligomers makes membranes leaky, leading to a Ca^2+^ imbalance, activation of Ca^2+^‐dependent enzymes (e.g., calpains/caspases), and mitochondrial dysfunction due to Ca^2+^ overload, including mPTP opening, depolarization, and pro‐apoptotic factor release. These processes promote a feed‐forward cycle in which Ca^2+^ overload and ROS reinforce each other, accelerating mitochondrial dysfunction and death signaling (Butterfield & Halliwell, 2019; Caroppi et al., 2009; De Felice et al., 2007; Di Scala et al., 2016; Foster, 2007; Görlach et al., 2015; Hewitt & Degnan, 2022; Lashuel et al., 2002; Lee et al., 2017; Li et al., 2013; Lin & Beal, 2006; Moreira et al., 2002; Quist et al., 2005; Talantova et al., 2013; Zhou et al., 2022).

Oligomers also compromise protein homeostasis (endosomal–lysosomal trafficking, the UPS, and autophagy), leading to the accumulation of misfolded proteins. The impaired clearance increases UPR/ER and oxidative stress. A consequence of sustained UPR/ER stress is a shift toward pro‐death signaling, increased trafficking/oligomer secretion, and decreased degradation capacity. Such a wide range of interconnected processes makes it challenging to assign a single dominant mechanism without specifying context. The toxicity effects of oligomers also depend on the cell type, subcellular localization of the oligomers, oligomer size/conformation, and lipid composition. For example, membrane disruption is strongly influenced by the lipid environment and may depend on the oligomer size, consistent with evidence that smaller oligomeric species may exhibit higher membrane solubility and toxicity than larger, lipid‐insoluble assemblies (Mrdenovic et al., 2019; Yang et al., 2017). Hence, defining which toxicity mechanisms are dominant requires mapping “which oligomer” (structure/size) to “which cellular target” (membranes, synapses, proteostasis, mitochondria) across timescales. Key knowledge gaps include resolving oligomer structural diversity in vivo, assigning structure–activity relationships to specific toxic pathways, pinpointing the main routes for oligomer spread/propagation, and integrating these mechanisms into models that reflect tissue‐level vulnerability and progression.

Addressing these gaps will require single‐entity, structure‐sensitive readouts capable of resolving heterogeneous oligomer populations and linking their physical states to specific cellular outcomes. New strategies for characterizing amyloid assemblies, including nanopore‐based tools, allow for highly sensitive detection of oligomer populations (Horne et al., 2025). Recent work has demonstrated large‐scale visualization and mapping of α‐synuclein oligomers in the Parkinson's disease brain, showing the capability for in situ detection of the oligomeric species, making them more accessible to study oligomer properties alongside their toxic effects (Andrews et al., 2025). Recent perspectives emphasize that oligomer structure and biological phenotype are tightly coupled, and that a key next step is resolving which in vivo oligomer populations (defined by PTMs and environment) drive specific disease phenotypes (Tang et al., 2025).

Amyloid oligomers are a critical target for understanding and treating amyloid‐related diseases. Still, their transient nature, conformational heterogeneity, and low in vivo abundance complicate their isolation and characterization. Hence, future progress will require the integration of structural biology, systems neuroscience, and clinical research, with careful consideration of the complex interplay of mechanisms that drive oligomer toxicity. A companion review discusses their structural diversity and the experimental techniques used to study them.

## AUTHOR CONTRIBUTIONS

**Magdalena I. Ivanova:** Conceptualization; investigation; writing – review and editing; writing – original draft. **Carmelo La Rosa:** Conceptualization; investigation; writing – review and editing; writing – original draft. **Ayyalusamy Ramamoorthy:** Conceptualization; investigation; writing – review and editing; funding acquisition; writing – original draft.

## CONFLICT OF INTEREST STATEMENT

The authors declare no conflicts of interest.

## Data Availability

Data sharing is not applicable to this article as no datasets were generated or analyzed during the current study.
